# Intranasal administration of acetylcholinesterase inhibitors

**DOI:** 10.1186/1471-2202-9-S3-S6

**Published:** 2008-12-10

**Authors:** Henry R Costantino, Alexis Kays Leonard, Gordon Brandt, Paul H Johnson, Steven C Quay

**Affiliations:** 1 Nastech Pharmaceutical Company Inc., 3830 Monte Villa Parkway, Bothell, Washington 98021, USA

## Abstract

This short review outlines the rationale, challenges, and opportunities for intranasal acetylcholinesterases, in particular galantamine. An *in vitro *screening model facilitated the development of a therapeutically viable formulation. *In vivo *testing confirmed achievement of therapeutically relevant drug levels that matched or exceeded those for oral dosing, with a dramatic reduction in undesired emetic responses. Intranasal drug delivery is an effective option for the treatment of Alzheimer's disease and other central nervous system disorders.

## Introduction

Intranasal (IN) delivery provides a viable and attractive option for administering various therapeutic agents [1]. Advantages of IN administration include a large surface area for delivery, rapid achievement of target drug levels, and avoidance of first pass metabolism; furthermore, this delivery route is noninvasive, maximizing patient comfort and compliance. In addition, IN dosing may facilitate transport of central nervous system (CNS) drugs into the brain [2-9], although this concept has not been universally established [10-14] and may depend on physicochemical properties of the drug [15-17]. Examples in the literature of CNS-related drugs given intranasally include opioids [18], benzodiazepines [19] and antimuscarinic agents [20], as well as acetylcholinesterase inhibitors [10,21-23].

## Acetylcholinesterase inhibitors for Alzheimer's disease

Acetylcholinesterase activity and inhibition have been the subject of studies for two decades, since the emergence of the cholinergic hypothesis, wherein deficits in learning, memory, and behavior are deemed to be associated with loss of cholinergic neurotransmission in the hippocampus and cortex [24]. Although various promising new therapeutic options are being vigorously pursued [25], acetylcholinesterase inhibitors remain the current front-line therapeutic approach to treatment of mild-to-moderate Alzheimer's disease (AD) [24,26]. AD is the most common form of disabling cognitive impairment in the elderly, and its increasing prevalence reflects a growing elderly population [27].

Examples of acetylcholinesterases used to treat AD include taurcine, rivastigmine, donepazil, and galantamine. Tacrine was the first acetylcholinesterase inhibitor approved for AD treatment [28], but this agent has been associated with some severe side effects, including hepatotoxicity, necessitating the research and development of newer inhibitors with greater specificity and higher potency. At present, commonly administered acetylcholinesterase inhibitors include rivastigmine, donepazil, and galantamine. Among these, galantamine possesses the dual mechanism of acetylcholinesterase inhibition and allosteric modulation of nicotinic acetylcholine receptors [29].

## Rationale for intranasal delivery of acetylcholinesterase inhibitors

Currently marketed acetylcholinesterase inhibitors are found entirely in oral dosage form. However, alternative routes, in particular IN administration, may provide benefits relative to oral dosing. For instance, the relatively low bioavailability of oral tacrine [30] has generated interest in delivery via various epithelial tissues, including the nasal route [31]. Similarly, the oral efficacy of physostigmine is limited because of low bioavailability, and investigations have focused on IN [10,21] and transdermal [32] delivery as alternatives to intravenous infusion. Transdermal physostigmine provides a mean absolute bioavailability of 36% as compared with only 3% for oral delivery in humans [32], and IN physostigmine may provide essentially complete bioavailability [21].

In addition to the avoidance of first pass metabolism, IN dosing also provides the potential for ameliorating adverse effects specific to the gastrointestinal (GI) tract. In the case of galantamine, GI-related side effects (for example, nausea and vomiting) are the adverse events that most commonly lead to discontinuation of treatment [33]. Moreover, the impact of galantamine on evacuative functions when it comes into contact with intestinal tissue has been described both *in vivo *and *in vitro *[34].

## Development of intranasal galantamine

Because of solubility and dose volume limitations, the commercially available form of galantamine, namely the hydrobromide salt, is not suitable for IN dosing. Therefore, an alternative drug formulation with a different, pharmaceutically acceptable counter cation, lactate, was developed [22]. The performance of galantamine-hydrobromide and galantamine-lactate was monitored using an *in vitro *epithelial tissue model and associated analyses [35]. Based on its increased solubility, low cytotoxicity, and high cell viability *in vitro*, galantamine-lactate represents a viable candidate for IN delivery.

Having developed a strategy to suitably increase the concentration of galantamine for IN delivery, the next step was to optimize its transepithelial permeation while retaining low toxicity [23]. Employing the same *in vitro *epithelial tissue model, various formulations containing permeation enhancers were screened via a design-of-experiments approach. Data collected during the *in vitro *screening phase included permeation, cytotoxicity, cell viability, and transepithelial electrical resistance (TER). The latter findings represent the integrity of the tight junctions between epithelial cells; reduction in TER corresponds with increased potential for paracellular, and hence overall, drug permeation. An optimal formulation was identified with low cytotoxicity, high cell viability, reduced TER, and enhanced permeation *in vitro*.

Various formulations were tested *in vivo *in rat [22,23]. There was a good correlation between the *in vitro *permeation rate and *in vivo *drug levels achieved, validating the utility of the epithelial tissue model. In the absence of permeation enhancers, the absolute oral and nasal bioavailability was approximately 22% to 23%; the incorporation of permeation enhancers approximately doubled the IN bioavailability (41%).

## Intranasal galantamine alleviates GI-related side effects.

In order to explore the hypothesis that IN dosing reduces GI-related side effects when compared with oral dosing, emetic responses were monitored for the oral versus IN galantamine formulations in a ferret model. The ferret was previously established to be a good model for emetic sensitivity [36]. The emetic study revealed a dramatic decrease in GI-related side effects when galantamine was administered by the IN route. During the first 4 hours after IN administration, only three emeses (retching events) were observed as compared with 34 with oral administration. The near absence of nausea, achieved despite similar or higher systemic exposure due to IN administration, further confirms that the observed emetic side effects associated with oral galantamine are caused by interactions in the GI tract and not systemic exposure to the drug.

## Conclusion

As shown using IN galantamine, IN delivery provides a viable and attractive option for administering various therapeutic drugs. An *in vitro *screening model facilitated the development of a therapeutically viable formulation, whereas i*n vivo *testing confirmed achievement of therapeutically relevant drug levels that matched or exceeded those for oral dosing, with a dramatic reduction in undesired emetic responses. The screening methods used show that IN drug delivery is an effective option for the treatment of AD and other CNS disorders.

## List of abbreviations used

AD: Alzheimer's disease; CNS: central nervous system; GI: gastrointestinal; IN: intranasal; TER: transepithelial electrical resistance.

## Competing interests

The authors declare that they have no competing interests.
